# Convolutional Neural Networks Can Predict Retinal Differentiation in Retinal Organoids

**DOI:** 10.3389/fncel.2020.00171

**Published:** 2020-07-03

**Authors:** Evgenii Kegeles, Anton Naumov, Evgeny A. Karpulevich, Pavel Volchkov, Petr Baranov

**Affiliations:** ^1^Department of Ophthalmology, The Schepens Eye Research Institute of Massachusetts Eye and Ear, Harvard Medical School, Boston, MA, United States; ^2^Genome Technologies and Bioinformatics Research Centre, Moscow Institute of Physics and Technology, Dolgoprudniy, Russia; ^3^Department of Information Systems, Ivannikov Institute for System Programming of the Russian Academy of Sciences, Moscow, Russia; ^4^National Research Center “Kurchatov Institute”, Moscow, Russia; ^5^Endocrinology Research Centre, Institute for Personalized Medicine, Moscow, Russia

**Keywords:** deep learning, convolutional neural networks, stem cells, retinal organoids, mouse embryonic stem cells

## Abstract

We have developed a deep learning-based computer algorithm to recognize and predict retinal differentiation in stem cell-derived organoids based on bright-field imaging. The three-dimensional “organoid” approach for the differentiation of pluripotent stem cells (PSC) into retinal and other neural tissues has become a major *in vitro* strategy to recapitulate development. We decided to develop a universal, robust, and non-invasive method to assess retinal differentiation that would not require chemical probes or reporter gene expression. We hypothesized that basic-contrast bright-field (BF) images contain sufficient information on tissue specification, and it is possible to extract this data using convolutional neural networks (CNNs). Retina-specific Rx-green fluorescent protein mouse embryonic reporter stem cells have been used for all of the differentiation experiments in this work. The BF images of organoids have been taken on day 5 and fluorescent on day 9. To train the CNN, we utilized a transfer learning approach: ImageNet pre-trained ResNet50v2, VGG19, Xception, and DenseNet121 CNNs had been trained on labeled BF images of the organoids, divided into two categories (retina and non-retina), based on the fluorescent reporter gene expression. The best-performing classifier with ResNet50v2 architecture showed a receiver operating characteristic-area under the curve score of 0.91 on a test dataset. A comparison of the best-performing CNN with the human-based classifier showed that the CNN algorithm performs better than the expert in predicting organoid fate (84% vs. 67 ± 6% of correct predictions, respectively), confirming our original hypothesis. Overall, we have demonstrated that the computer algorithm can successfully recognize and predict retinal differentiation in organoids before the onset of reporter gene expression. This is the first demonstration of CNN’s ability to classify stem cell-derived tissue *in vitro*.

## Introduction

The differentiation of pluripotent stem cells (PSC) using a three-dimensional “organoid” approach has become the strategy of choice to recapitulate the development of the retina, brain, inner ear, intestine, pancreas, and many other tissues *in vitro* (McCauley and Wells, 2017). This technique allows to reproduce the process of normal development and does not require any exogenous stimulation of developmental pathways and genetic modification of the cells used (Eiraku et al., 2011; Meyer et al., 2011). Indeed hundreds of studies confirm that retinal organoids, differentiated from mouse or human pluripotent cells, show a unique resemblance to native tissue architecture, cell specification and sub-specification, function, and transcriptional profile (Hallam et al., 2018; Cowan et al., 2019). This demonstrates the robustness of the technology and makes it highly attractive for potential translation to the clinic as a source of high-quality retinal neurons for transplantation (Decembrini et al., 2014) or as a platform for the screening of new therapeutics (Baranov et al., 2017).

The process of the differentiation itself is stochastic, which causes the quantity of retinal differentiation to vary a lot even among organoids within one batch—not to say when different cell lines are used (Hiler et al., 2015; Hallam et al., 2018; Cowan et al., 2019). The current approach to select retinal tissue for further growth and maturation is based on subjective morphological observation and features visible with bright-field imaging: lamination of the neuroepithelium, adjacent pigment epithelium areas, *etc*., and/or on the expression of fluorescent reporter constructs driven by retina-specific promoters. These reporters allow to assess the differentiation on different stages of retinal development: from early eye field-specific genes [Pax6-GFP mESCs (Völkner et al., 2016) and Rx-GFP mESCs (Eiraku et al., 2011)] to terminal retinal cell types as rods Nrl-GFP miPSCs (Ueda et al., 2018), Six6 (Sluch et al., 2018), or Rx (Nakano et al., 2012) for early optic vesicles, Brn3a (Sluch et al., 2015) for retinal ganglion cells, Crx (Nakano et al., 2012) for photoreceptors, or Nrl (Phillips et al., 2018) for human rods.

The use of fluorescent reporters is a “gold standard”—it is a sensitive, specific, and easily quantifiable method to assess retinal differentiation (Vergara et al., 2017), although it cannot be used in cell manufacture for transplantation or to model inherited diseases due to genome modification. The manual selection under the microscope with bright-field imaging is limited in throughput and the classification criteria can be subjective, resulting in high variability between observers. This puts its limitations on the further transition of this technology “from the bench to bedside.” Here we tried to address this issue by developing an automated non-invasive method which can predict retinal differentiation based on bright-field images of retinal organoids on the early stage of their development using artificial intelligence.

Machine learning has been evolving rapidly during the last decades. This is mostly due to the increase in accessible computational power and the ability to generate and store massive amounts of data. Nowadays, one of the most actively developing branches of artificial intelligence is deep learning, which was able to outperform the best conventional machine learning algorithms in multiple fields including speech and image recognition (LeCun et al., 2015). This technology was inspired by the principles which lay in cognition and data processing by the brain. In simple understanding, the biological neuron is receiving information from other neurons, combines it, and transmits a modified signal to the next pool of neurons. In general, the artificial neuron works in a similar way: it receives inputs from the group of neurons, combines them with some weights for each input, and transmits the result to the next set of neurons using some non-linear function. So, each artificial neuron itself can be interpreted as a function, which gets a vector of inputs from neurons from the previous layer and returns some value (activation) which is being transmitted to the next layer. The neural network usually contains several layers of these neurons connected together, starting from the input layer and finishing with the output layer which returns the result. The general task for supervised learning is to find optimal weights for each neuron in the network to minimize an error between the value predicted by the program and the value which was assigned before the training (e.g., ground truth label for classification or some score for regression task).

This approach showed itself to be extremely effective in solving multiple tasks such as speech recognition, computer vision (LeCun et al., 2015), processing of medical and biological data (Ching et al., 2018), etc. For the analysis of images (or any data which has local adjacency structure), the special type of neural networks was developed—convolutional neural networks (CNN). This type of neural network has a few so-called convolutional layers in the beginning of the learning process, which allows to find relationships between spatially adjacent parts of the image for the dimensionality reduction and extraction of features. This approach has found a lot of applications in multiple fields of biology and medicine. For example, for diagnosis of diabetic retinopathy based on fundus imaging (Gulshan et al., 2016) and for skin cancer classification (Esteva et al., 2017), and recently it was proven effective to predict the very early onset of PSC differentiation (Waisman et al., 2019) and the quality of retinal pigment epithelium (RPE) differentiation in a two-dimensional setting (Schaub et al., 2020). Being inspired by the success that this approach showed on the prediction of spontaneous differentiation of PSCs with basic bright-field imaging used as a source of information, we hypothesized that basic-contrast bright-field images contain sufficient information on tissue specification, and it is possible to extract it using convolutional neural networks. In this study, we decided to test the ability of CNN to: (1) recognize early retinal differentiation in organoids; and (2) predict retinal differentiation in individual organoids before the onset of the expression of the eye field-specific reporters—for instance, Rx.

To predict early retinal differentiation, we utilized a transfer learning approach: CNN is being pretrained on the ImageNet classification dataset (Deng et al., 2009) containing more than 10 million images which are split into more than 20,000 classes. This approach allows to transfer the ability of a pretrained network to extract low-level features from natural images and focus more on high-level features from the target dataset during the training. Such a trick helps to achieve desirable results using lower amounts of training data and have been proven useful for the analysis of biological images (Ching et al., 2018).

## Materials and Methods

### mES Cell Culture

mES reporter cell line RxGFP has been used in this study (RIKEN; Eiraku et al., 2011). The cells were cultured in the mES medium (Supplementary Table S1), fed every other day, and passaged at 70–80% confluence on a cell culture-treated T-75 flask coated with 1% Matrigel (Corning) solution for 1 h. For replating or seeding for retinal organoid formation, the cells were dissociated using 0.25 Trypsin solution (Gibco) for 7 min on 37°C in a CO_2_ incubator.

### Retinal Differentiation

**Figure 1:**
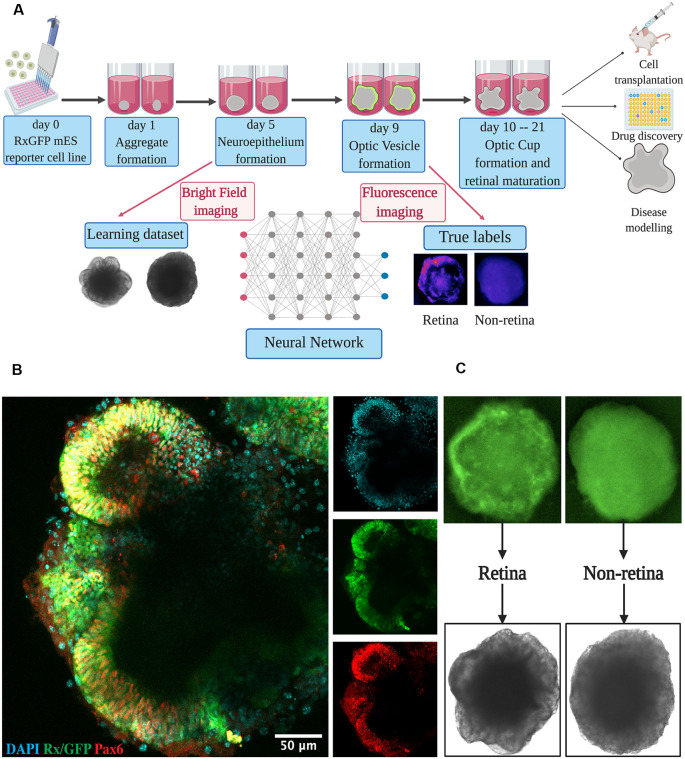
Retinal differentiation. **(A)** Experimental outline: the organoids were imaged on day 5 using bright-field and on day 9 using fluorescent microscopy. Fluorescent images were used to assign true labels and bright-field ones for feeding neural network. This figure was created with BioRender.com. **(B)** Confocal image of retinal organoid on day 9 of retinal differentiation. Staining was performed for early retina-specific markers: Rx and Pax6. **(C)** Representative organoids from retinal and non-retinal classes. Different patterns in fluorescent images reflect the difference in bright-field ones.

Retinal differentiation was performed as was shown before, with minor modifications (Perepelkina et al., 2019). The protocol is outlined in Figure 1A. The RxGFP mES cells were dissociated from the flask with 0.25 trypsin and seeded in a differentiation medium (OV; Supplementary Table S1) on a 96-well U-bottom polystyrene plate (Greiner) at a cell density of 3,000 cells per well in 50 μl of the media. The cells were fed with 50 μl of OV supplemented with 1% Matrigel (Corning) on day 1 of differentiation. Additional feeding with 50 μl of OV with 0.5% Matrigel was performed on day 5 of differentiation. Further medium change was performed with OC media starting from day 9.

### Automated Imaging

Both bright-field and fluorescent images of the organoids have been taken using the EVOS fl Auto microscope. For bright-field imaging, the plates were scanned with a 4× phase-contrast objective on day 5 of differentiation, with fine autofocus function. As each organoid is seeded separately in a separate well of a 96-well plate, each image contained no more than one organoid.

### Immunohistochemistry and Confocal Imaging

Ten organoids from each batch were collected and fixed with 4% PFA for 20 min at room temperature (RT). Prior to staining, they were blocked with a blocking buffer for 1 h at RT. Staining with primary antibodies (Santa-Cruz anti-Rx antibody #SC-79031 and Hybridoma Bank anti-PAX6 antibody #AB528427) was performed overnight at +4°C in staining buffer. On the next day, after washing with a wash buffer (Supplementary Table S2), secondaries were applied overnight at +4°C. After staining with antibodies and washing, the organoids were stained with 4′,6-diamidino-2-phenylindole for 10 min at RT and mounted on concavity slides (Lab Scientific). Confocal images were taken using a Leica SP5 confocal microscope.

### Classification Criteria for Fluorescent Images

The discrimination between retinal and non-retinal organoids for the purpose of assigning ground truth labels was based primarily on the expression of the Rx-GFP reporter, which is a very specific marker for early retinal progenitor cells (Medina-Martinez et al., 2009; Zagozewski et al., 2014). The criteria took into account the brightness of the reporter, localization, and pattern of the retinal area.

**Figure 2:**
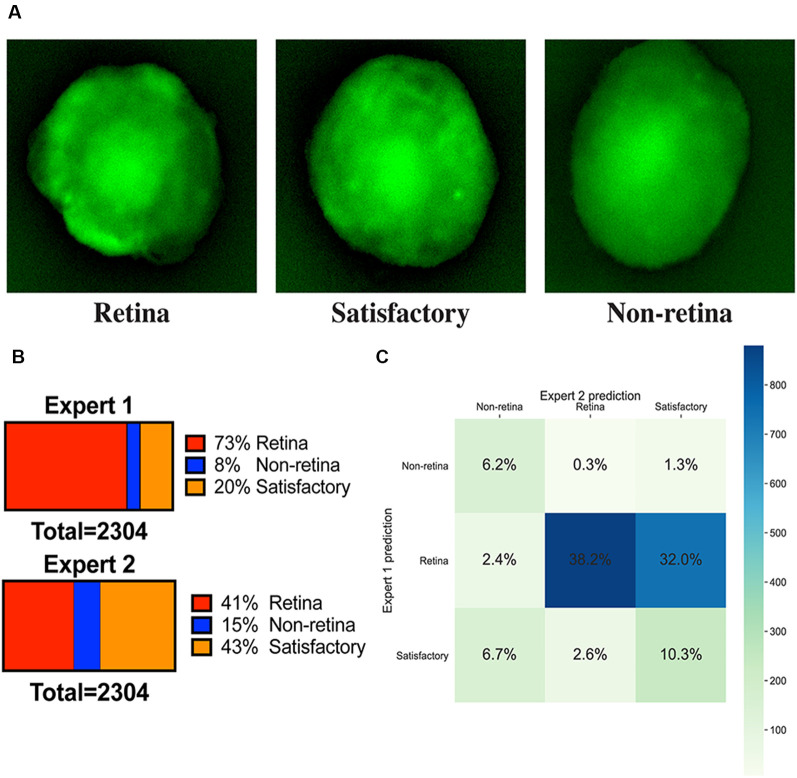
Image annotations. **(A)** Fluorescent images of representative organoids from each class which experts have classified to “retina,” “non-retina,” and “satisfactory.” **(B)** Ratios of labels assigned by two experts for the training dataset. **(C)** Summary of ratios for different classes which can be assigned after combining the votes from two experts.

We have sorted organoids based on the fluorescent images on day 9 into three groups: “retina,” “non-retina,” and “satisfactory.” The following criteria were utilized (Figure 2):

- The retinal organoids should have bright fluorescence or localized fluorescent retina-like structures.
- A satisfactory organoid should have sparse or scattered fluorescence pattern without clearly separable retinal areas.
- A non-retinal organoid should not be fluorescent or have uniformly distributed green background fluorescence.

### Classification Criteria for Bright-Field Images

For sorting organoids on day 6 using bright-field images, the following criteria were defined:

- Retina—distinct layer-like (neuroepithelium) transparent areas on the periphery of the organoids
- Non-retina—uniform cellular aggregate without distinct transparent areas

### Dataset Preparation and Images Preprocessing for Training the Network

The initial dataset (1,209 images in total) was split into three parts: the training one (64% of total), the validation (16% of total), and the test one (20% of total). The training and validation datasets were used for architecture and parameter selection. The test dataset was used only for the final validation of the best neural network after the whole process of parameter tuning and architecture selection is completed.

Before feeding the images to neural networks, we implemented a few preprocessing steps. First, we find the position of the organoid on an image and crop it out using Python OpenCV script based on blob detection. This is a very simple and straightforward approach for object detection. It works best if the target object is significantly darker or brighter than the background as it is based on automated thresholding (Otsu method). This is exactly the case for retinal organoids—they are significantly darker than the background and have pretty contrast borders. Thus, we found the algorithm to work very efficiently. Furthermore, it does not require any manual parameter adjustments, except for the average organoid size which stays stable, if the same quantity of cells is used for seeding in the beginning of differentiation.

We also applied Gaussian normalization to the images and augmented them with random horizontal and vertical flips, rotations, width and height shifts, and zoom transformations. Proportionally more transformations were applied to the non-retina class images in order to balance the number of images used for CNN training. Additional information on augmentation parameters can be found in the “Supplementary Extended Methods” section.

### Interpretation of CNN Output and Threshold Selection

The neural network takes some piece of data as an input, i.e., image, and is designed to predict the probability for it to be retinal—value between 0 and 1. This is done in the following way.

The network consists of small abstract units called neurons; each of those has several inputs (like axons/dendrites in real neurons). Each dendrite of each neuron has its own value called weight, and each neuron itself has its own value called bias. When a neuron gets some numerical values to its inputs, it multiplies them with the corresponding weights, sums them up, adds bias, and applies to the result some non-linear function (usually called activation function). The resulting value is sent to the output.

The neurons are aggregated into groups called layers. The inputs of the neurons of the first layer are attached to the pixels of the input image. The inputs of the neurons from any internal layer are attached only to the outputs from the neurons in the preceding layers. The last layer consists only of one neuron—its output value is interpreted as the probability of the organoid to be a retinal one. The way to organize the neurons and the layers is called the architecture of the network.

Initially, the weights and the biases of the neurons are taken randomly. While the network gets training images, it tries to predict their classes, evaluates the results using true classes of the images, and adjusts the weights and the biases of its neurons using a backpropagation algorithm.

Therefore, after processing the image, CNN returns a value from 0 to 1, which can be interpreted as a probability for this organoid to belong to the “retina” class. Thus, the threshold should be selected to make the final prediction: organoids with scores higher than the threshold would be considered “retinal,” and with lower—“non-retinal.”

We determined a threshold by maximizing the value of sensitivity * specificity [true positive rate * (1- false positive rate)] on the training dataset. This approach helps to improve both the sensitivity and the specificity of the classifier, which can be affected by the imbalance between classes.

### Selection of the Best CNN Architecture and Cross-Validation

For our task, we selected four convolutional neural networks with different architectures, which showed themselves effective on ImageNet competitions and in multiple biological applications (Esteva et al., 2017; Waisman et al., 2019): VGG19 (Simonyan and Zisserman, 2014), ResNet50v2 (He et al., 2016), DenseNet121 (Huang et al., 2017), and Xception (Chollet, 2017). All of these CNNs were initially pretrained on the ImageNet dataset (Deng et al., 2009).

For the selection of the best network, 10-folds cross-validation was used: the training dataset was split into 10 non-overlapping subsets. On each step of the process, the network is training on nine out of these 10 subsets and then uses the last subset for validation. Each subset is used for validation once. So, this allows to perform statistical tests for CNN performance comparison.

### Hyperparameters Tuning and Training of the Networks

The training was performed on the training dataset, and multiple hyperparameters have been optimized using the validation dataset (learning rate, set of frozen layers, dropout rate of the last layer, etc.). Additional information on the actual values of the hyperparameters used for each CNN can be found in the “Supplementary Extended Methods” section and Supplementary Table S3. Also, as we are using transfer learning approach, only the few last layers of the CNN are trained. The number of these layers depends on the architecture chosen and should be also considered as a hyperparameter.

### Assessment of CNN Performance

There are multiple approaches available to measure the performance of classifiers, including accuracy, F1 score, receiver operating characteristic-area under the curve (ROC-AUC), Mathews correlation coefficient (MCC), and many others.

The simplest and the most intuitive score is “accuracy”—the number of correct guesses divided by the total number of samples in the dataset. Additional metrics are “precision”—number of objects which were correctly predicted as positive divided by the total number of objects selected as positive, and “recall” or “true positive rate”—number of objects which were correctly predicted as positive divided by the total number of positive objects in the initial dataset. The accuracy shows how many selected objects are really the correct ones, and the recall shows how many of the relevant objects the algorithm was able to pick up. As precision and recall cannot be optimized separately, metrics which take into account both these values are usually used. The F1 score is a harmonic mean of precision and recall. However, all of these scores have some drawbacks, especially for imbalanced data, as both classes are treated equally and changes in a wrongly predicted minor class do not have a high impact on the score.

Alternatively, MCC can be calculated—the value which shows how well the predictions of the classifier and the true labels are correlated. One of the advantages of this metric is that it can be very sensitive even when classes are imbalanced.

Another option is using ROC-AUC score—the area under the ROC curve (true positive rate vs. false positive rate at different threshold values). It is the “gold standard” for binary classification with neural networks. It has a very meaningful interpretation: this value shows the probability of a randomly selected object from the “retina” class to have a higher score than a random object from the “non-retina” class. So, for a classifier that assigns labels randomly, the score would be 0.5, and for the perfect algorithm, it would be equal to 1. Therefore, this score can be considered as the measure of order which the classifier provides. Thus, we chose the ROC-AUC score as the main measure of performance for our CNN.

## Results

### Retinal Differentiation and Initial Annotation of the Collected Images by Experts

For dataset collection, approximately 3,000 retinal organoids were differentiated and analyzed. For the training of our neural network and annotating the dataset, we collected bright-field and fluorescent images for each organoid on day 5 and day 9 of differentiation, respectively (Figures 1A,C). On day 9, in most organoids, distinct optic vesicle areas could be observed. In Figure 1B, a confocal image of retinal organoids on day 9 of differentiation is presented. Retina-like planar structures are formed on the periphery of the organoid; these areas are also positive for retina-specific markers Pax6 and Rx. As Rx is known to be an essential transcription factor for retinal development (Zagozewski et al., 2014), we chose its expression at day 9 to be a ground truth indication for early retinal differentiation.

All fluorescent images were collected on day 9 and pooled together, filtered to get rid of pictures with poor quality, anonymized, and offered to two independent experts for sorting in three groups: (1) good retina (Figure 1C, left; Figure 2A, left); (2) satisfactory retina (Figure 2A, center); and (3) not retina (Figure 1C, right; Figure 2A, right). The classification criteria are stated in the “Materials and Methods” section. The proportions of each class for each expert are provided in Figure 2B, and the cumulative distribution of organoids after classification is summarized in Figure 2C.

For our network, we stated the two-class classification problem: we asked the program to extract features which would distinguish high-quality organoids from bad ones based only on bright-field images. To do that, we generated the training dataset by assigning to organoids with label “retina” only if both experts put this organoid in class “retina” and “non-retina” if at least one suggested it to be non-retinal. Classes “retina/non-retina,” “retina/satisfactory,” and “satisfactory/satisfactory” were not used for training the network. The resulting dataset consisted a total of 1,209 bright-field images, with the proportion of classes at 73 vs. 27% for retina and non-retina, respectively. As each organoid is seeded in a separate well and they are developing independently, we consider each of them to be an independent biological replicate.

### Selection of the Best CNN Architecture

**Figure 3:**
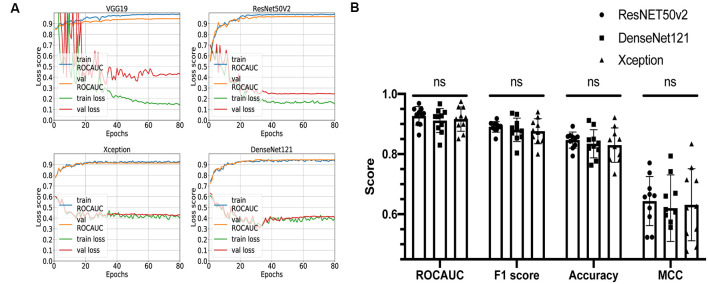
Comparison of different convolutional neural network (CNN) architectures. **(A)** Loss curves and receiver operating characteristic-area under the curve (AUC) training curves for VGG19, ResNET50v2, DenseNet121, and Xception. **(B)** Comparison summary of three different CNNs using 10-fold cross-validation. The mean AUC scores were 0.93 ± 0.03 vs. 0.91 ± 0.04 vs. 0.92 ± 0.04 (*P* = 0.3) for ResNET50v2, DenseNet121, and Xception, respectively; the mean F1 scores were 0.89 ± 0.02 vs. 0.88 ± 0.04 vs. 0.88 ± 0.04 for ResNET50v2, DenseNet121, and Xception, respectively; the mean accuracy scores were 0.85 ± 0.03 vs. 0.83 ± 0.05 vs. 0.83 ± 0.06 for ResNET50v2, DenseNet121, and Xception, respectively; the mean Matthews correlation coefficients were 0.64 ± 0.08 vs. 0.62 ± 0.11 vs. 0.63 ± 0.12 for ResNET50v2, DenseNet121, and Xception, respectively. Each dot on the graph corresponds to one cross-validation step. ns, not significant (*P*-value > 0.05 on Friedman statistical test).

Four networks based on different architectures (VGG19, ResNet50v2, Xception, and DenseNet121) have been trained and validated on the dataset. The learning curves are shown in Figure 3A. All networks were successfully trained, but the VGG19-based classifier shows signs of overfitting: loss score on validation dataset is significantly higher than on training dataset; so, for further comparison, we decided to keep only ResNet50v2-, Xception-, and DenseNet121-based CNNs.

The remaining three networks were run through 10-fold cross-validation, and for each step, ROC-AUC score, optimal thresholds, F1, MCC, and accuracy scores were calculated (Figure 3B). The mean AUC scores were 0.93 ± 0.03 vs. 0.91 ± 0.04 vs. 0.92 ± 0.04 (*P* = 0.3) for ResNet50v2, DenseNet121, and Xception, respectively; the mean F1 scores were 0.89 ± 0.02 vs. 0.88 ± 0.04 vs. 0.88 ± 0.04 (*P* = 0.6) for ResNet50v2, DenseNet121, and Xception, respectively; the mean accuracy scores were 0.85 ± 0.03 vs. 0.83 ± 0.05 vs. 0.83 ± 0.06 (*P* = 0.6) for ResNet50v2, DenseNet121, and Xception, respectively; and the mean Matthews correlation coefficients were 0.64 ± 0.08 vs. 0.62 ± 0.11 vs. 0.63 ± 0.12 for ResNet50v2, DenseNet121, and Xception, respectively. All of the networks show similar results, and no significant difference has been found using the Friedman test (analog of Wilcoxon test when three or more samples are compared). So, we can conclude that all of these CNNs can potentially be utilized for solving our task. However, the Xception- and DenseNet121-based CNNs had a noticeable variation of the loss score for the different validation steps of cross-validation (Supplementary Figure S1). Also, we noticed that ResNet50v2 had the smallest standard deviation among other classifiers for each metric (Figure 3B); therefore, at this step, we selected this CNN.

### Convolutional Neural Network Can Predict Early Retinal Differentiation

**Figure 4:**
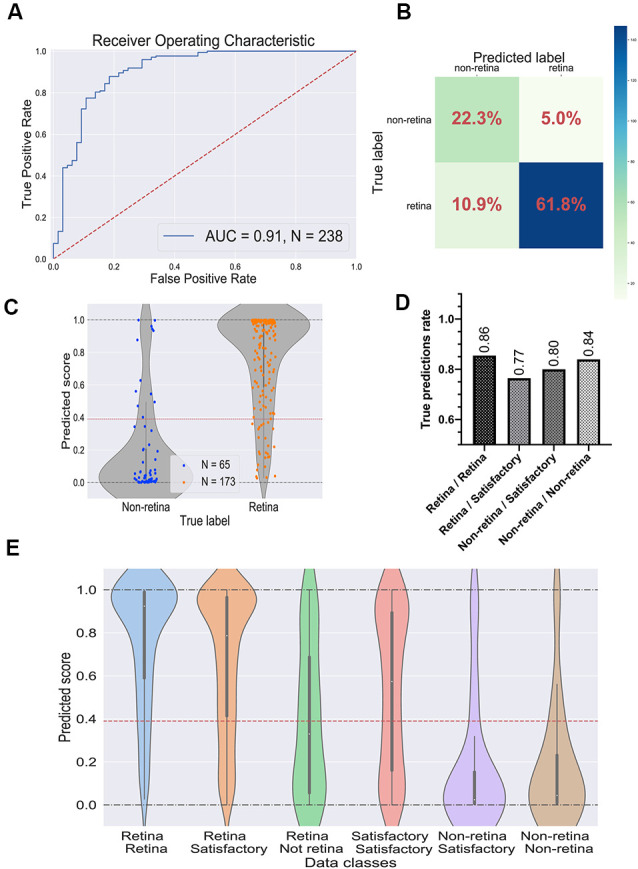
Performance of the best convolutional neural network (CNN) on the test dataset. **(A)** Receiver operating characteristic (ROC) curve for the selected CNN on the test dataset. ROC-area under the curve value equals 0.91. **(B)** Confusion matrix for selected CNN. Values in the squares represent percentages of true negative, false positive, false negative, and true positive predictions. Color LUT shows the absolute number of images in each group. **(C)** Prediction scores for the test dataset; each dot represents a single organoid; the red line represents the threshold value for the classifier. **(D)** True prediction rates for each class of organoids with the CNN classifier. **(E)** Violin plots on all possible classes of organoids which can be assigned by combining the votes from two experts. The white dot in the center of each plot represents the median of the distribution; the boxes and the bars represent the first quartile and the upper/lower adjacent value, respectively; the red line is a classifier’s threshold.

To evaluate the performance of the selected CNN, we utilized the test dataset which was not used during the training and parameter tuning process. The ROC curve is shown in Figure 4A and the confusion matrix in Figure 4B. For this dataset, the predictor showed the ROC-AUC score to be 0.91 (Figure 4A), accuracy—0.84, F1 score—0.89, and Matthews correlation coefficient—0.63. Despite a significant imbalance between the retinal and the non-retinal classes, the classifier was able to reach 0.85 sensitivity and 0.82 specificity scores on the test dataset. This indicates that augmentation and threshold selection allowed to efficiently tackle the imbalance problem.

The prediction scores for every single image and the threshold are shown in Figure 4C. As expected, the retinal and the non-retinal organoids are “condensed” at the corresponding values: 0 for non-retina and 1 for the retina; so, the model clearly can separate these two types of organoids.

Then, we decided to have a look at the performance of the model on different classes, which were obtained after combining the experts’ annotations. The true prediction rates for each class are presented in Figure 4D. Expectedly, the best performance the model shows on organoids which came from “sure” classes: retina/retina and non-retina/non-retina, meaning that the CNN is more likely to be mistaken where experts are also less sure about the labels. Moreover, in Figure 4E; the distributions of the prediction scores are shown for each class. Again, retina/retina and non-retina/non-retina classes are clearly separated. Moreover, organoids from retina/satisfactory class, which were not used for training and validation, also were in most cases correctly attributed by the network to the retina class, although the median of the distribution is shifted from 1, showing that the program gets confused more often than on “retina/retina” class, which is also consistent with the result shown in Figure 4D.

Interestingly, the predictor could not separate organoids from the retina/non-retina group, which can be concluded from the fact that the median of the scores is located close to the threshold: it can be interpreted as CNN is working almost as a random predictor for organoids from this group. Organoids from satisfactory/satisfactory class also can be poorly distinguished, but the median is shifted toward the retinal class, which is being in accordance with the criteria that we used for this class.

To identify the image areas and features that are used by the CNN, we utilized SHapley Additive exPlanations (SHAP) value approach (Lundberg and Lee, 2017). We noticed that the border of the organoids and, more specifically, the retina-like neuroepithelium loops on the periphery are zones of interest for the CNN (Supplementary Figure S2).

### CNN Outperforms Human Classifier on Prediction of Retinal Differentiation

To compare the CNN performance with the human-based classifier, we asked four independent experts to assign the labels “retina” and “non-retina” for organoids from the test dataset. The criteria for this classification can be found in the “Materials and Methods” section.

**Figure 5:**
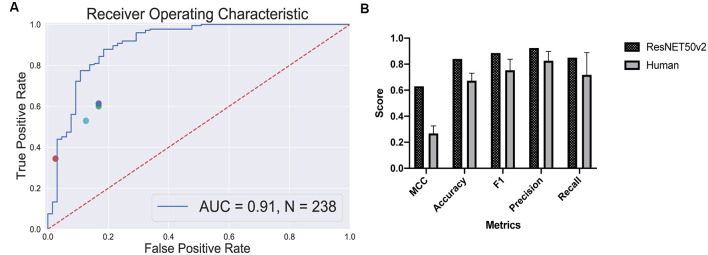
Human-based classifier vs. CNN-based classifier. **(A)** The receiver operating characteristic curve for the convolutional neural networks (CNN); the area under the curve score for this classifier is 0.91. Each dot represents a single human expert predicting organoids to be from “retina” or “non-retina” class based on bright-field images. **(B)** Metrics comparison for human-based classifier and CNN-based. CNN showed better results on all the metrics that we measured: 0.63 vs. 0.27 ± 0.06 Matthews correlation coefficient for CNN and human, respectively; 0.84 vs. 0.67 ± 0.06 accuracy for CNN and human, respectively; 0.89 vs. 0.75 ± 0.09 F1 score for CNN and human, respectively; 0.92 vs. 0.83 ± 0.07 precision for CNN and human, respectively; 0.85 vs. 0.72 ± 0.17 recall for CNN and human, respectively.

True positive rates and false positive rates for each expert are plotted on the classifier’s ROC curve (Figure 5A). The CNN is clearly outperforming a human in distinguishing retinal differentiation on the early stage of differentiation. Different metrics for the comparison are provided in Figure 5B. On average, a human expert has an accuracy of 0.67 ± 0.06, while CNN has an accuracy of 0.84.

The more striking difference gives the comparison of a Matthews correlation coefficient which takes into account class disbalance: 0.63 vs. 0.27 ± 0.06 for Matthews correlation coefficient for CNN and human, respectively.

## Discussion

Retinal organoid cultures have a great potential to model human disease and development and as source of retinal neurons for transplantation or platform for therapeutics testing. The remaining challenges, highlighted in RPE transplantation studies, include high variability between different cell lines (Leach et al., 2016), scaled production with automation or other approaches (Regent et al., 2019), and lack of cGMP-compatible non-invasive readouts for the assessment of differentiation during the development process (Schaub et al., 2020). The translational success of regenerative therapies based on iPSCs-derived RPE (Mandai et al., 2017; da Cruz et al., 2018) is largely due to the development of strategies to overcome these issues. In this study, we attempted to address the latter for retinal 3D organoids.

There are two distinct, non-mutually exclusive approaches to characterize and identify the differentiating cell with non-invasive imaging techniques. The classic strategy is to define the exact features and the thresholds that are characteristics of a particular cell type. This approach is based on our understanding on how the cell looks *in vivo*: this was demonstrated in decades of RPE differentiation studies *in vitro* (Thumann et al., 2013), where pigmentation, cell shape, and autofluorescence can be quantified and compared to the pre-set quality criteria thresholds (da Cruz et al., 2018; Schaub et al., 2020). The evolution of this approach involves better understanding of the thresholds as well as introduction of new imaging techniques that can detect new features—multispectral fluorescent and non-fluorescent imaging, optical coherence tomography (Browne et al., 2017; Capowski et al., 2019), and others. An alternative strategy is machine learning that is also highly dependent on the modality by which the information is collected. However, the information is processed in a different way: it does not require any predefined criteria for assessment—the CNN learns how to find and extract the most relevant features from the data by itself, provided that the program “has seen” enough samples to learn it from. Machine learning becomes particularly valuable when there are multiple criteria and definitions or when they are not very well established. In this case, the training of computer algorithm occurs with the help of experts, who would classify or rank the training set of images, i.e., cats vs. dogs (Krizhevsky et al., 2012), early vs. late diabetic retinopathy (Pratt et al., 2016), etc. This technology becomes extremely powerful when it is possible to use an orthogonal approach, “other modality,” to make a decision on what class the object belongs to: molecular profile (Waisman et al., 2019) or functional response (Schaub et al., 2020). This is the exact case of retinal differentiation using 3D organoid strategy: there are limited accurate criteria distinguishing good retinal organoids and bad ones with BF imaging, especially on the early stage of their development, although the availability of reporter cell lines allows to determine retinal differentiation with high accuracy. Here we showed that such discrimination is possible with a convolutional neural network which could predict early retinal differentiation based only on universal bright-field imaging.

One of the major questions in the area of deep learning is the role of individual features in image recognition. It is not clear which parts of the image are most important for the algorithm to classify an object. This issue of potential unpredictability becomes more important when the action is solely based on artificial intelligence decision. Also, by extracting individual features that are most important in predicting cell behavior, it may be possible to identify novel biological processes and identify the actual determinants of retinal formation in the embryoid bodies. By using SHAP value approach, we were able to show the importance of translucent neuroepithelium-like structures in decision-making (Supplementary Figure S2), although we were not able to show the actual causality of these structures in the decision-making process.

The program clearly outperformed the experts in a classification task (Figure 5) and was able to predict eye field induction better than a human performing a morphological observation of organoid with bright-field microscopy: 0.84 vs. 0.67 ± 0.06 accuracy for CNN vs. human, respectively. This additionally illustrates that the criteria for the selection of retinal organoids at this stage are subjective. Furthermore, the good performance of the CNN-based classifier shows that the morphology of the organoids, even on a very early stage, contains sufficient information to predict retinal differentiation, and the program can extract this information.

Moreover, the approach does not require any complicated imaging, fluorescent reporters, or dyes for analysis; so, it can be easily implemented in almost any laboratory or manufacturing setting. Therefore, our method offers a robust and universal non-invasive approach for the assessment of retinal differentiation.

As we have stated the problem as a classification task, we assume from the beginning that there should be some threshold which would distinguish retinal and non-retinal organoids. However, there are many organoids which are “on the border”—these organoids we called “satisfactory” organoids; these are hard to separate in two distinct classes with the single fluorescent reporter. Moreover, for different applications, different thresholds may be needed: for example, for disease or development modeling, the quality of the organoid should be prioritized to get the proper physiology and morphology of the retina, but for cell production, the yield may be a priority and a lower threshold can be applied for enrichment. Moreover, for drug discovery, using retinal organoids can be problematic as the amount of retinal differentiation varies between different organoids, and having a method to grade organoids can be helpful to interpret the assay results. Therefore, having an ability to select a threshold according to the task can be rather important for different applications. Thus, one of the further directions to be considered is a statement of the regression problem for grading retinal organoids. This would significantly expand the possible applications of the approach. However, this task would require a reliable quantification method to assign “ground truth” values for the network training. One of the possible metrics which can be utilized is not only the simple quantification of total fluorescence in the organoid (Vergara et al., 2017) if using fluorescent reporters but also the localization and the shape of retina-like structures might be important parameters which should be taken into account, as well as the physiological and the metabolic state of the retinal neurons (Browne et al., 2017).

We have used mouse embryonic stem cells with Rx reporter in this work. Using this gene expression as a specific indicator of eye field induction, we were able to predict differentiation using CNN. We consider that the approach that we established can be easily translatable not only to other mouse reporter cell lines but also for human organoids. This is due to the fact that the method relies only on the morphology of the organoids during development, and Sasai’s differentiation protocol has been shown to be effective on human embryonic stem cells (Nakano et al., 2012). Moreover, here are multiple retina-related human PSC reporter cell lines available, which target different cell types and differentiation stages: Six6 (Sluch et al., 2018) and Rx (Nakano et al., 2012) for early optic vesicles, Brn3a (Sluch et al., 2015) for retinal ganglion cells, Crx (Nakano et al., 2012) for photoreceptors, or Nrl (Phillips et al., 2018) for rods specifically. Therefore, our approach with training the CNN to predict the differentiation can also be utilized for human cells and possibly for later differentiation stages. However, to achieve the best results on human cells, additional training for mouse-pretrained neural network may be required to adjust to possible morphological differences between mouse and human organoids.

Moreover, as we have shown that CNN can accurately predict retinal differentiation based only on simple bright-field images of the organoids, we suppose that not only microscope images can be utilized for the CNN training. For example, probably this approach can be incorporated in the large-particle flow cytometer machines as an alternative to fluorescence.

## Data Availability Statement

The datasets generated for this study are available on request to the corresponding author.

## Author Contributions

EK, AN, and PB conceived the experiments. EK designed and performed the differentiation experiments, interpreted the results, and wrote the manuscript with the help of AN. AN and EAK trained the neural networks and performed the comparison of different CNN architectures. EK and PB developed an idea and annotated fluorescent and bright-field images. All the authors discussed the experiments and the manuscript. EAK, PV, and PB provided funding for this study. PB revised and corrected the manuscript. All the authors read and approved the final version of the manuscript.

## Conflict of Interest

The authors declare that the research was conducted in the absence of any commercial or financial relationships that could be construed as a potential conflict of interest.

## References

[B1] Baranov P. Lin H. McCabe K. Gale D. Cai S. Lieppman B.. (2017). A novel neuroprotective small molecule for glial cell derived neurotrophic factor induction and photoreceptor rescue. J. Ocul. Pharmacol. Ther. 33, 412–422. 10.1089/jop.2016.0121 28441076 PMC5911694

[B2] Browne A. W. Arnesano C. Harutyunyan N. Khuu T. Martinez J. C. Pollack H. A.. (2017). Structural and functional characterization of human stem-cell-derived retinal organoids by live imaging. Invest. Ophthalmol. Vis. Sci. 58, 3311–3318. 10.1167/iovs.16-20796 28672397 PMC5495152

[B3] Capowski E. E. Samimi K. Mayerl S. J. Phillips M. J. Pinilla I. Howden S. E.. (2019). Reproducibility and staging of 3D human retinal organoids across multiple pluripotent stem cell lines. Development 146:dev171686. 10.1242/dev.171686 30567931 PMC6340149

[B4] Ching T. Himmelstein D. S. Beaulieu-Jones B. K. Kalinin A. A. Do B. T. Way G. P.. (2018). Opportunities and obstacles for deep learning in biology and medicine. J. R. Soc. Interface 15:20170387. 10.1098/rsif.2017.0387 29618526 PMC5938574

[B5] Chollet F. (2017). “Xception: deep learning with depthwise separable convolutions,” in Proceedings of the 2017 IEEE Conference on Computer Vision and Pattern Recognition (CVPR), 1800–1807.

[B6] Cowan C. S. Renner M. Gross-Scherf B. Goldblum D. Munz M. Krol J. (2019). Cell types of the human retina and its organoids at single-cell resolution: developmental convergence, transcriptomic identity and disease map. SSRN Electr. J. [Epub ahead of print]. 10.2139/ssrn.3438371 PMC7505495 32946783

[B7] da Cruz L. Fynes K. Georgiadis O. Kerby J. Luo Y. H. Ahmado A.. (2018). Phase 1 clinical study of an embryonic stem cell-derived retinal pigment epithelium patch in age-related macular degeneration. Nat. Biotechnol. 36, 328–337. 10.1038/nbt.4114 29553577

[B8] Decembrini S. Koch U. Radtke F. Moulin A. Arsenijevic Y. (2014). Derivation of traceable and transplantable photoreceptors from mouse embryonic stem cells. Stem Cell Reports 2, 853–865. 10.1016/j.stemcr.2014.04.010 24936471 PMC4050344

[B9] Deng J. Dong W. Socher R. Li L.-J. Li K. Fei-Fei L. (2009). “ImageNet: a large-scale hierarchical image database,” in 2009 IEEE Conference on Computer Vision and Pattern Recognition, 248–255.

[B10] Eiraku M. Takata N. Ishibashi H. Kawada M. Sakakura E. Okuda S.. (2011). Self-organizing optic-cup morphogenesis in three-dimensional culture. Nature 472, 51–58. 10.1038/nature09941 21475194

[B11] Esteva A. Kuprel B. Novoa R. A. Ko J. Swetter S. M. Blau H. M.. (2017). Dermatologist-level classification of skin cancer with deep neural networks. Nature 542, 115–118. 10.1038/nature21056 28117445 PMC8382232

[B12] Gulshan V. Peng L. Coram M. Stumpe M. C. Wu D. Narayanaswamy A.. (2016). Development and validation of a deep learning algorithm for detection of diabetic retinopathy in retinal fundus photographs. 316, 2402–2410. 10.1001/jama.2016.17216 27898976

[B13] Hallam D. Hilgen G. Dorgau B. Zhu L. Yu M. Bojic S.. (2018). Human-induced pluripotent stem cells generate light responsive retinal organoids with variable and nutrient-dependent efficiency. Stem Cells 36, 1535–1551. 10.1002/stem.2883 30004612 PMC6392112

[B14] He K. Zhang X. Ren S. Sun J. (2016). “Identity mappings in deep residual networks,” in Computer Vision—ECCV 2016, eds Leibe B. Matas J. Sebe N. Welling M. (Cham: Springer International Publishing), 630–645.

[B15] Hiler D. Chen X. Hazen J. Kupriyanov S. Carroll P. A. Qu C.. (2015). Quantification of retinogenesis in 3D cultures reveals epigenetic memory and higher efficiency in IPSCs derived from rod photoreceptors. Cell Stem Cell 17, 101–115. 10.1016/j.stem.2015.05.015 26140606 PMC4547539

[B16] Huang G. Liu Z. van der Maaten L. Weinberger K. Q. (2017). “Densely connected convolutional networks,” in Proceedings of the 2017 IEEE Conference on Computer Vision and Pattern Recognition (CVPR), 2261–2269.

[B17] Krizhevsky A. Sutskever I. Hinton G. E. (2012). “ImageNet classification with deep convolutional neural networks,” in Proceedings of the Advances in Neural Information Processing Systems, 1097–1105.

[B18] Leach L. L. Croze R. H. Hu Q. Nadar V. P. Clevenger T. N. Pennington B. O.. (2016). Induced pluripotent stem cell-derived retinal pigmented epithelium: a comparative study between cell lines and differentiation methods. J. Ocul. Pharmacol. Ther. 32, 317–330. 10.1089/jop.2016.0022 27182743 PMC5911695

[B19] LeCun Y. Bengio Y. Hinton G. (2015). Deep learning. Nature 521, 436–444. 10.1038/nature14539 26017442

[B20] Lundberg S. M. Lee S. I. (2017). “A unified approach to interpreting model predictions,” in Proceedings of the Advances in Neural Information Processing Systems, 4766–4775.

[B21] Mandai M. Watanabe A. Kurimoto Y. Hirami Y. Morinaga C. Daimon T.. (2017). Autologous induced stem-cell-derived retinal cells for macular degeneration. N. Engl. J. Med. 376, 1038–1046. 10.1056/NEJMoa1608368 28296613

[B22] McCauley H. A. Wells J. M. (2017). Pluripotent stem cell-derived organoids: using principles of developmental biology to grow human tissues in a dish. Development 144, 958–962. 10.1242/dev.140731 28292841 PMC5358106

[B370] Medina-Martinez O. Amaya-Manzanares F. Liu C. Mendoza M. Shah R. Zhang L.. (2009). Cell-autonomous requirement for Rx function in the mammalian retina and posterior pituitary. PLoS One 4, 1–7. 10.1371/journal.pone.0004513 19229337 PMC2641000

[B23] Meyer J. S. Howden S. E. Wallace K. A. Verhoeven A. D. Wright L. S. Capowski E. E.. (2011). Optic vesicle-like structures derived from human pluripotent stem cells facilitate a customized approach to retinal disease treatment. Stem Cells 29, 1206–1218. 10.1002/stem.674 21678528 PMC3412675

[B24] Nakano T. Ando S. Takata N. Kawada M. Muguruma K. Sekiguchi K.. (2012). Self-formation of optic cups and storable stratified neural retina from human ESCs. Cell Stem Cell 10, 771–785. 10.1016/j.stem.2012.05.009 22704518

[B25] Perepelkina T. Kegeles E. Baranov P. (2019). Optimizing the conditions and use of synthetic matrix for three-dimensional *in vitro* retinal differentiation from mouse pluripotent cells. Tissue Eng. Part C Methods 25, 433–445. 10.1089/ten.tec.2019.0053 31195897

[B26] Phillips M. J. Capowski E. E. Petersen A. Jansen A. D. Barlow K. Edwards K. L.. (2018). Generation of a rod-specific NRL reporter line in human pluripotent stem cells. Sci. Rep. 8:2370. 10.1038/s41598-018-20813-3 29402929 PMC5799252

[B27] Pratt H. Coenen F. Broadbent D. M. Harding S. P. Zheng Y. (2016). Convolutional neural networks for diabetic retinopathy. Proc. Comput. Sci. 90, 200–205. 10.1016/j.procs.2016.07.014

[B28] Regent F. Morizur L. Lesueur L. Habeler W. Plancheron A. Ben M’Barek K.. (2019). Automation of human pluripotent stem cell differentiation toward retinal pigment epithelial cells for large-scale productions. Sci. Rep. 9:10646. 10.1038/s41598-019-47123-6 31337830 PMC6650487

[B29] Schaub N. J. Hotaling N. A. Manescu P. Padi S. Wan Q. Sharma R.. (2020). Deep learning predicts function of live retinal pigment epithelium from quantitative microscopy. J. Clin. Invest. 130, 1010–1023. 10.1172/JCI131187 31714897 PMC6994191

[B30] Simonyan K. Zisserman A. (2014). “Very deep convolutional networks for large-scale image recognition,” in Proceedings of the 3rd International Conference on Learning Representations, ICLR 2015—Conference Track, 1–14. http://arxiv.org/abs/1409.1556.

[B31] Sluch V. M. Davis C. H. O. Ranganathan V. Kerr J. M. Krick K. Martin R.. (2015). Differentiation of human ESCs to retinal ganglion cells using a CRISPR engineered reporter cell line. Sci. Rep. 5:16595. 10.1038/srep16595 26563826 PMC4643248

[B32] Sluch V. M. Chamling X. Wenger C. Duan Y. Rice D. S. Zack D. J. (2018). Highly efficient scarless knock-in of reporter genes into human and mouse pluripotent stem cells *via* transient antibiotic selection. PLoS One 13:e0201683. 10.1371/journal.pone.0201683 30496180 PMC6264506

[B33] Thumann G. Dou G. Wang Y. Hinton D. R. (2013). “Chapter 16—Cell biology of the retinal pigment epithelium,” in Retina, Fifth Edition, ed Stephen J. Ryan (Elsevier), 401–414.

[B34] Ueda K. Onishi A. Ito S. Nakamura M. Takahashi M. (2018). Generation of three-dimensional retinal organoids expressing rhodopsin and S- and M-cone opsins from mouse stem cells. Biochem. Biophys. Res. Commun. 495, 2595–2601. 10.1016/j.bbrc.2017.12.092 29274337

[B35] Vergara M. N. Flores-Bellver M. Aparicio-Domingo S. McNally M. Wahlin K. J. Saxena M. T.. (2017). Three-dimensional automated reporter quantification (3D-ARQ) technology enables quantitative screening in retinal organoids. Development 144, 3698–3705. 10.1242/dev.146290 28870990 PMC5675442

[B36] Völkner M. Zschätzsch M. Rostovskaya M. Overall R. W. Busskamp V. Anastassiadis K.. (2016). Retinal organoids from pluripotent stem cells efficiently recapitulate retinogenesis. Stem Cell Reports 6, 525–538. 10.1016/j.stemcr.2016.03.001 27050948 PMC4834051

[B37] Waisman A. La Greca A. Möbbs A. M. Scarafía M. A. Velazque N. L. S. Neiman G.. (2019). Deep learning neural networks highly predict very early onset of pluripotent stem cell differentiation. Stem Cell Reports 12, 845–859. 10.1016/j.stemcr.2019.02.004 30880077 PMC6449871

[B38] Zagozewski J. L. Zhang Q. Pinto V. I. Wigle J. T. Eisenstat D. D. (2014). The role of homeobox genes in retinal development and disease. Dev. Biol. 393, 195–208. 10.1016/j.ydbio.2014.07.004 25035933

