# Supplementary material for: Convolutional Neural Networks Can Predict Retinal Differentiation in Retinal Organoids
Source: Front Cell Neurosci. 2020 Jul 3;14:171. doi: 10.3389/fncel.2020.00171 (PMC7350982; doi:10.3389/fncel.2020.00171)
Supplement: Supplementary file 1 [file Data_Sheet_1.docx]

**Supplementary Figure 1.** *Learning curves for cross-validation for three tested architectures.* Loss curves (A, C, D ) and ROCAUC scores (B, D, F) for cross-validation on training dataset. Each line on the graph corresponds to single step of cross-validation. Three CNN architectures were tested: ResNet50v2 (A, B), Xception (C, D), DenseNet121 (E, F)

**
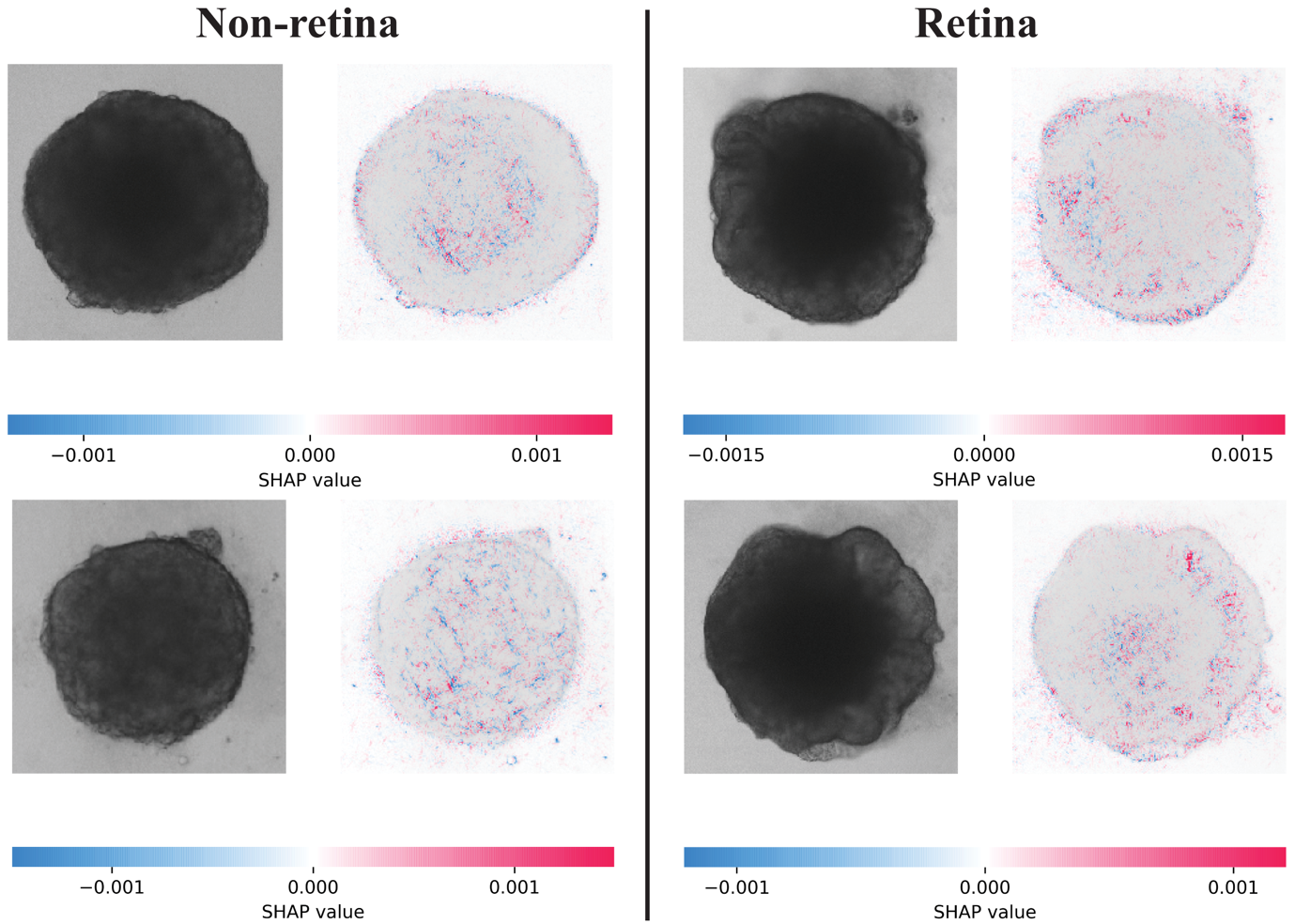
**

**Supplementary Figure 2.** *SHAP values to explain the importance of different parts of the image for the Network prediction.* These images represent SHAP values for different examples from test dataset. Blue and red dots correspond to different SHAP values in accordance with LUT under each picture. Positive values mean that this regions shifts the decision towards “retina” class, negative values – towards “non-retina”.

**Suppl. Table 1** Culture medium composition

**Suppl. Table 2** Buffers composition

|  | **MES** | **OV** | **OC** |
| --- | --- | --- | --- |
| DMEM/F12 solution (Gibco) | 500ml | 500ml | 500ml |
| Sodium pyruvate 100 mM (Sigma) | 5.5 ml | 5.2 ml | 5.2 ml |
| Non-essential amino acids (Gibco) | 5.5 ml | 5.2 ml | 5.2 ml |
| Antibiotic-Antimycotic (Gibco) | 5.5 ml | 5.2 ml | 5.2 ml |
| Chemically defined lipid concentrate (Gibco) | - | 5.2 ml | 5.2 ml |
| Fetal Bovine Serum (Gibco) | 55 ml | 7.6 ml | - |
| LIF | 1000 UI/ml | - | - |
| Insulin Transferrin Selenium-Ethanolamine (ITS-X 100X) (Gibco) | - | 1 ml | - |
| N-Acetyl-L-Cystein (NAC)  0.5mM stock solution | - | 1.25 ml | 1.25 ml |
| b-Mercaptoethanol 16M (Sigma) | 4 ul | 4 ul | 4 ul |
| NS21 supplement | - | - | 10 ml |
| Forskolin | - | 10 uM | 10 uM |
| Retinoic Acid (Sigma) | - | - | 0.5 uM |
| Taurine (Sigma) | - | - | 1 mM |
| Retinoic Acid Receptor Antagonist (Tocris) | - | 1 uM | - |

| **Blocking Buffer** | |
| --- | --- |
| PBS | 1x |
| Goat serum | 10 % |
| Triton-X | 0.25 % |
| Tween-20 | 0.25 % |
| Sodium Citrate | 0.1 % |
| Bovine Serum Albumin | 1 % |
| **Staining Buffer** | |
| PBS | 1x |
| Triton-X | 0.25 % |
| Tween-20 | 0.25 % |
| Bovine Serum Albumin | 1 % |
| **Washing Buffer** | |
| PBS | 1x |
| Triton-X | 0.1 % |
| Tween-20 | 0.1 % |

**Suppl. Table 3** Hyperparameters for different CNNs

|  | Xception | ResNet50V2 | VGG19 | DenseNet121 |
| --- | --- | --- | --- | --- |
| Initial LR | 0.05 | 0.005 | 0.005 | 0.005 |
| Reduce LR patience(epochs) | 5 | 3 | 5 | 6 |
| Dropout rate(last two layers) | 0.1 | 0.8 | 0.1 | 0.1 |

***Extended Methods***

*CNN training setting*

All CNNs used for this study we constructed from one of the for known architectures (VGG19, DenseNet121,  ResNet50V2 and Xception) without the last layer used for predictions in their original setting. All training and testing procedures as well as mentioned CNN architectures were implemented with Keras library.

The dimensions of input images were: 224x224x3.

On the top of these networks we put several additional layers, subsequently:  average pooling layer, dropout layer, 512-output dense layer, batch Normalization layer, ReLU activation, dropout layer, 2-output dense layer with sigmoid activation. Both dropout layers share the same dropout rate parameter for within one network.

All networks used binary cross entropy for optimisation. On plateau learning rate (LR) reduction was utilized by all the networks with minimal LR of 1e-09 and reduce factor of 0.5. The original training lasted for 80 epochs, the monitored quality used for LR reduction and best model saving was ROCAUC score on validation dataset. Each cycle of 10-fold cross-validation was run on 40 epoches, with the quality for monitor being ROCAUC score on the train dataset.

The hyperparameters specific for different CNNs are listed in the Suppl. Table 3.

The CNNs used the following optimizers:

Xception: SGD with Nesterov momentum. The momentum parameter is 0.03

ResNet50V2: SGD with Nesterov momentum. The momentum parameter is 0.002

VGG19: Adam with beta_1 = 0.9 and beta_2 = 0.99

DenseNet121: SGD with Nesterov momentum. The momentum parameter is 0.002

*Transfer Learning setting*

All CNNs were taken from Keras library with the weights obtained by the training on ImageNet dataset. During the training on our dataset the following layers were frozen:

DenseNet121: from 0 to 4.

VGG19: from 0 to 10.

ResNet50V2: from 0 to 4.

Xception: from 0 to 24.

*Augmentation parameters*

During the training we used with Keras ImageDataGenerator for on the fly augmentation with the random horizontal and vertical flips, rotations in the range of 5 degrees, vertical and horizontal shifts with the range of 0.1 and zoom transformations with the range of 0.1. We also used samplewise centering and normalization.

*SHAP values analysis*

To interpret CNN predictions we used the approach based on Shapley values – SHAP (Lundberg and Lee 2017). This approach works best when it is possible to "forget" about some features in the sample. Thats cannot be done for CNNs in a straightforward manner like for decision trees or for linear model. To overcome this difficulty one needs to define a background — the "default" values of pixels of the image. After this is done the “forgetting” about the feature means assigning the corresponding value from the background.

To calculate SHAP values for our task we utilized SHAP DeepExplainer: for each image we wanted to interpret shap values were calculated 100 times, each time with different background taken randomly from train dataset. The resulting shap images are the averages of these 100 shap values.
